# Implementing neuroimaging and eye tracking methods to assess neurocognitive development of young infants in low- and middle-income countries

**DOI:** 10.12688/gatesopenres.12951.2

**Published:** 2019-08-27

**Authors:** Laura Katus, Nathan J. Hayes, Luke Mason, Anna Blasi, Samantha McCann, Momodou K. Darboe, Michelle de Haan, Sophie E. Moore, Sarah Lloyd-Fox, Clare E. Elwell

**Affiliations:** 1 Great Ormond Street Institute of Child Health, University College London, London, London, WC1N 1EH, UK; 2 GKT School of Medical Education, King’s College London, London, UK; 3 Centre for Brain and Cognitive Development, Birkbeck University of London, London, UK; 4 Department of Medical Physics and Biomedical Engineering, University College London, London, UK; 5 Department of Women and Children’s Health, King's College London, London, UK; 6 Medical Research Council Unit The Gambia at the London School of Hygiene and Tropical Medicine, The Gambia, The Gambia; 7 Great Ormond Street Hospital for Children, London, UK; 8 Department of Psychology, University of Cambridge, Cambridge, UK

**Keywords:** neurocognitive development, infancy, fNIRS, EEG, eye tracking, low-and middle-income countries (LMIC), global health

## Abstract

Infants and children in low- and middle-income countries (LMICs) are frequently exposed to a range of environmental risk factors which may negatively affect their neurocognitive development. The mechanisms by which factors such as undernutrition and poverty impact development and cognitive outcomes in early childhood are poorly understood. This lack of knowledge is due in part to a paucity of objective assessment tools which can be implemented across different cultural settings and in very young infants. Over the last decade, technological advances, particularly in neuroimaging, have opened new avenues for research into the developing human brain, allowing us to investigate novel biological associations. This paper presents functional near-infrared spectroscopy (fNIRS), electroencephalography (EEG) and eye tracking (ET) as objective, cross-cultural methods for studying infant neurocognitive development in LMICs, and specifically their implementation in rural Gambia, West Africa. These measures are currently included, as part of a broader battery of assessments, in the Brain Imaging for Global Health (BRIGHT) project, which is developing brain function for age curves in Gambian and UK infants from birth to 24 months of age. The BRIGHT project combines fNIRS, EEG and ET with behavioural, growth, health and sociodemographic measures. The implementation of these measures in rural Gambia are discussed, including methodological and technical challenges that needed to be addressed to ensure successful data acquisition. The aim is to provide guidance to other groups seeking to implement similar methods in their research in other LMICs to better understand associations between environmental risk and early neurocognitive development.

## Introduction

Children growing up in low- and middle-income countries (LMICs) are at increased risk of compromised neurocognitive development due to exposure to a range of adverse environmental factors. Poor developmental outcomes have been associated with a variety of interacting risk factors, including undernutrition, poor sanitation and increased rates of infectious disease, and limited social interaction ( Grantham-McGregor *et al.*, 2007; Joseph *et al.*, 2014; Walker *et al.*, 2007). An estimated 165 million children world-wide are undernourished and stunted in growth, the largest proportion of which live in countries in sub-Saharan Africa ( UNICEF, 2013). In addition to high rates of stunting in this region, the rate of young children who fail to achieve age-appropriate developmental levels of cognitive and social emotional functioning has been estimated to lie between 38–60% ( McCoy *et al.*, 2016). This indicates that currently there is a significant but largely unexplored co-occurrence between early-life adversity and cognitive functioning, affecting a vast number of children. Impairments in cognitive domains such as memory, attention, and language can severely impede children’s everyday functioning and academic achievement, with long-term detrimental effects reaching well into adulthood. Despite the scope of the problem, our understanding of how environmental risk factors affect early brain and cognitive development is poor and limits our ability to develop appropriate interventions to address these outcomes.

The first 1000 days of life, spanning conception to approximately 24 months of age, is a crucial period of infant development. During this time, the foundations of sensory, cognitive and social development are established, allowing individuals to thrive in later life. Development during this time is not only critical but also especially vulnerable to adverse environmental factors ( Andersen, 2003; Rice & Barone, 2000). A thorough understanding of infant brain and cognitive development during this early period is therefore crucial when seeking to understand the impact of environmental factors on later developmental outcomes. Only by understanding typical developmental trajectories is it possible to establish standards against which the efficacy of interventions can be evaluated.

‘One limiting factor in studying neurodevelopmental trajectories in LMICs is the limited availability of assessment tools that can offer objective, mechanistic insight into brain development, with proven validity across cultures. Much neurodevelopmental research to date has been based on behavioural assessments. While these assessments have vastly advanced this field of research, they have some limitations when used in longitudinal, multicentre cross-cultural research. When used during the first year or life, the predictive validity of behavioural assessments has been found to be reduced, especially when used to assess at-risk populations, such as infants with very low birthweight ( Hack *et al*., 2005) and infants with familial risk of autism ( Elsabbagh & Johnson, 2010; Yirmiya & Charman, 2010). While behavioural responses sufficient to indicate altered developmental pathways are often only evident towards the second year of life, significantly earlier changes in brain functional and anatomical specialisation have been found in the first months of life in at-risk populations ( Bishop, 2007; Elsabbagh *et al*., 2012; Lloyd-Fox *et al*., 2017). This indicates the potential for an earlier window of detection and implementation of intervention than behavioural measures commonly allow. When implemented after infants’ first birthday, neurobehavioural assessments become much more accurate and reproducible, but also time consuming, limiting their potential for routine use in clinical practice. While behavioural assessments have and continue to offer valuable insights in neurodevelopmental research, neuroimaging and eye tracking can provide additional insights; implementing these during the first months of life holds potential to uncover early correlates of later behavioural outcome, and importantly can inform our understanding of underlying mechanisms in the developing brain that might give rise to either typical or atypical outcomes.’

Technological advances, especially in the field of neuroimaging have yielded several tools which are able to detect correlates of brain functioning; either by measuring electrical activity generated by the brain (e.g. electroencephalography (EEG), magnetoelectroencephalography (MEG)) or the consequent haemodynamic/metabolic responses (e.g. positron emission tomography (PET), magnetic resonance imaging, (MRI), functional near infrared spectroscopy (fNIRS)). Many of these neuroimaging techniques, which are well established in adults, have specific attributes which restrict or prevent their use in infants. PET requires the use of radioisotopes, whilst MRI and MEG require the participant to remain very still, usually swaddled or restrained during sleep or sedation. Furthermore, while pioneering research is currently being undertaken with techniques such as MRI in resource poor urban global health projects ( Turesky *et al*., 2019), its high cost and low portability makes its application challenging in rural areas and non-clinical settings. In contrast, fNIRS and EEG rely on headgear that can be rapidly fitted and is well tolerated by infants of all age groups. Above that, both techniques rely on hardware that is portable and relatively low cost. EEG and fNIRS can be regarded as complementary methods, which allow us to assess both temporal and spatially localised features of the neuronal and subsequent haemodynamic response. EEG is a non-invasive technique that, using lightweight headgear, records electrical activity of cortical pyramidal neurons at scalp level. Data is acquired from a number of electrodes placed at different locations across the head. While the conduction of the signal through the brain, scalp and skull leads to a reduced spatial resolution compared to fMRI or fNIRS, the temporal resolution of EEG recordings at ~1ms is excellent. fNIRS is a relatively novel technique, which has been adapted and optimised for the study of infant development over the past decade ( Gervain *et al.*, 2011; Lloyd-Fox *et al.*, 2010; Taga *et al.*, 2017). fNIRS is a non-invasive optical neuroimaging technique that can measure spatially localised cortical brain function. Infants wear lightweight headgear which facilitates the delivery to, and detection of, near-infrared light from the head. Changes in near-infrared light intensity are a correlate of changes in haemodynamics and oxygenation arising from localised neuronal activity in the underlying cortical tissue ( Villringer & Chance, 1997).

In addition to infant-friendly neuroimaging measures such as fNIRS and EEG, eye tracking (ET) is another method shown to be able to capture neurodevelopmental changes throughout infancy. ET involves recording a participant’s point of gaze, either to a screen or to a naturalistic environment. fNIRS, EEG and ET all offer high levels of objectivity as they can be used to directly measure low-level, neurocognitive responses, making them well suited for use in cross- cultural assessment of a broad range of cognitive domains. This paper discusses the implementation of the three technologies in The Gambia, West Africa as part of the ongoing Brain Imaging for Global Health (BRIGHT) project ( The BRIGHT project). The BRIGHT project longitudinally tracks two infant cohorts from birth to two years of age, one in a rural village in The Gambia (n = 223), and the other in the UK (n = 61). Data collection began in June 2016 and in ongoing, with all assessments being completed by July 2020.

The Gambian arm of the BRIGHT project is carried out at the Medical Research Council Gambia (MRCG) Unit at the London School of Hygiene and Tropical Medicine’s Keneba, field station ( www.mrc.gm; www.ing.mrc.ac.uk). The field station is located in the West Kiang region, a three-hour drive from the coastal capital of Banjul. MRCG Keneba has conducted research and provided healthcare in this region since the 1950’s and over the years built a close relationship with the local community. The majority of people within the West Kiang region are of the Mandinka ethnic group, and the community is primarily Islamic. The population are largely subsistence farmers, and many aspects of health and behaviour are strongly influenced by the pronounced seasonality in this region ( Hennig *et al.*, 2017). Undernutrition, especially in infants and young children, remains a significant public health problem, with recent rates of stunting (height for age <2 SD below the WHO reference) and underweight (weight for height <-2SD) averaging at 22 and 30%, respectively ( Nabwera *et al.*, 2017). One of the main goals of the BRIGHT protocol is to capture information to assess the impact of nutritional deficiency and growth stunting on brain development.

fNIRS, EEG and ET have been implemented in high-income countries for many years, and several groups have now begun to implement these methods in populations in LMICs, including Bangladesh ( Jensen *et al*., 2019; Perdue *et al*., 2019; Xie *et al*., 2018) Brazil ( Shephard *et al*., 2019) and India ( Wijeakumar *et al*., 2019) In this paper we will discuss how our group successfully implemented these three measures in rural Gambia. We will first provide a description of the fNIRS, EEG and ET set up that we use as part of the BRIGHT project protocol, before discussing some specific adaptations to our protocol that were found necessary for use in The Gambia. We will then describe some specific challenges that were encountered that might bear relevance to other groups working in similar settings. It is hoped, that this will provide encouragement and guidance for other researchers working in LMICs who are looking to incorporate measures of neurocognitive development in their work. Our aim is to help to establish a wider network of global health research groups implementing similar methods across different sites to enable a cross cultural and populations studies of the effect of early life adversity on infant brain development.

## Methods implemented in BRIGHT project: fNIRS, EEG and ET

### Functional Near Infrared Spectroscopy (fNIRS)

**Figure 1.**
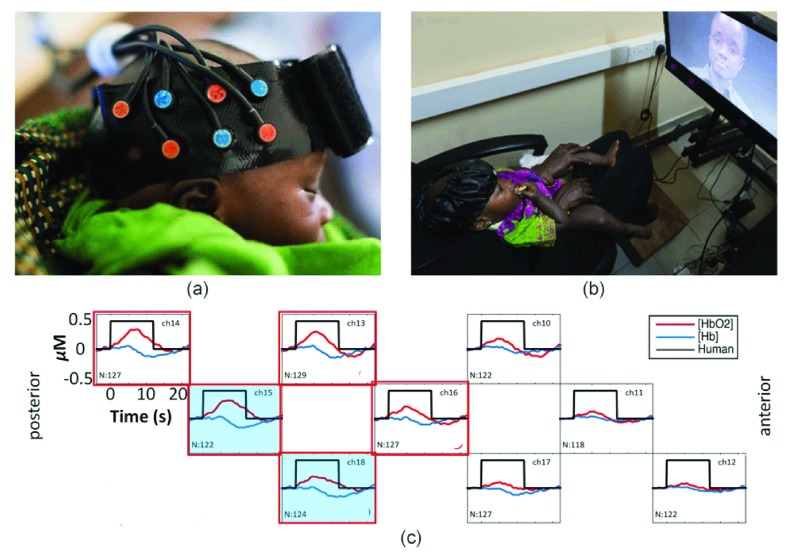
Set up and output of one BRIGHT fNIRS study. ( **a**) A sleeping infant wearing the fNIRS headband holding sources (red) and detectors (blue) in a similar set up to the data shown in the lower panel. ( **b**) Set up of fNIRS study in The Gambia for awake infants. Infant is seated on parent’s lap wearing the fNIRS headgear while attending to stimuli on the screen. ( **c**) Typical haemodynamic responses to an auditory stimulus across nine channels distributed across infants’ right temporal lobe. Responses are averaged across a group of 129 one-month-old infants (from the first wave of data collection in the BRIGHT project) showing localised increases in HbO _2_ (red) and a corresponding decrease in HHb (blue) in response to auditory stimulation; red frames illustrate a significant increase of HbO _2_, blue shading indicates a significant decrease in HHb. N, number of infants with valid data for each channel; ch, channel number. Image copyright: Gates Foundation ( **a**), Ian Farrell ( **b**).

In the BRIGHT project, fNIRS data is collected using the NTS optical imaging system (Gowerlabs Ltd. London, Everdell *et al.*, 2005), which emits near-infrared light at the 780nm and 850nm wavelengths, allowing the simultaneous measurement of changes in oxyhaemoglobin (HbO _2_) and deoxyhaemoglobin (HHb). During data acquisition, infants wear custom-built silicone headbands which hold in place multiple source and detector optodes and fibres ( Figure 1a). The headgear is carefully aligned with anatomical landmarks of the head during placement, to ensure the optodes measure underlying brain activity in the same cortical regions across infants. Since an initial pilot study in 2013, the use of fNIRS has been implemented to study infant brain development between birth and 24 months of age In The Gambia ( Begus *et al.*, 2016; Lloyd-Fox *et al.*, 2017; Lloyd-Fox *et al.*, 2014; Papademetriou *et al.*, 2014). This work has culminated in an extended, longitudinal, fNIRS protocol implemented for the current project. In the BRIGHT project, the majority of tasks take the form of passive visual or auditory paradigms which are presented to infants on a screen using standardised distances and light levels, or as audio, at standardised volumes. At 1 month of age infants are tested asleep using auditory stimuli only ( Figure 1a), while at all other age points infants are tested awake while seated on their parent’s lap ( Figure 1b). Figure 1c shows a typical haemodynamic response pattern across the different channels within a source-detector array. As neuronal activity increases, localised cerebral blood flow increases, producing an increase in HbO _2_ and a decrease (washout) of HHb. Thus, by measuring the changes of HbO _2_ and HHb over multiple sources and detectors, robust inferences can be drawn about the functional activation of different cortical regions.

**Figure 2.**
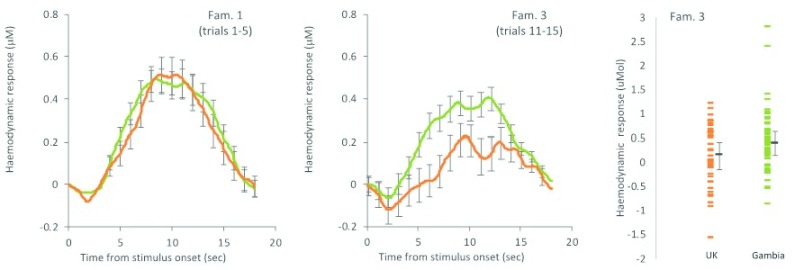
BRIGHT Project Habituation and Novelty Detection (HaND) study. Left: haemodynamic HbO _2_ eight-month-old group response to repeated stimuli. Right: individual amplitudes. Participants: UK (orange), N=42; Gambia (green), N=60. For familiarisation trial (Fam.) 3, lower = better. High data quality allows for a clear differentiation between cohorts as indicated by non-overlapping error bars. (Adapted from figures in Lloyd-Fox *et al*., 2019).

The fNIRS studies implemented in the BRIGHT project assess a range of cognitive functions, including social cognition ( Lloyd-Fox *et al.*, 2017), habituation and novelty detection (HaND, Lloyd-Fox *et al.*, 2019), working memory ( Begus *et al.*, 2016) as well as functional connectivity between cortical areas (for an example of the adaptations to stimuli used in these tasks please see sub-section on Stimuli below). Data quality, as indicated by retention rates of data sets after rejection of noisy or corrupted data, has been similar (and in some cases better) to previous developmental studies in high-income countries. Figure 2 shows data collected from the HaND paradigm within the BRIGHT cohort at 8 months of age in The Gambia and in the UK (for details on this paradigm see Lloyd-Fox *et al.*, 2019). The responses indicate differential changes in the haemodynamic response, with infants in The Gambia showing an attenuated habituation rate to the presentation of repeating stimuli relative to infants in the UK. Furthermore, the high data quality allows for comparison of data at the individual infant level, which is important for tracing longitudinal developmental trajectories.

Data retention varied across age points and paradigm, similarly to previously published fNIRS studies ( Lloyd-Fox *et al.*, 2010), but importantly was found to be broadly similar across the parallel sites. Within the recently analysed data for the HaND paradigm for example, 60–75% of the data collected was valid at 1, 5 and 8 months across the UK and The Gambia. Despite having established fNIRS as a viable measure in this setting ( Lloyd-Fox *et al*., 2014), the larger scope of the BRIGHT project posed some additional challenges which are discussed in the following section.

***Maintenance of the system.*** Although the NTS optical imaging system only rarely requires maintenance, certain factors related to BRIGHT measures in The Gambia, including very heavy use (with peak use reaching up to four infants tested per day for seven days a week over a two year period), dust, heat and variable humidity, power surges and outages were likely to increase the maintenance requirements during the BRIGHT project. It was therefore important to establish a process by which any necessary repairs could be carried out by locally based staff to prevent delays in data collection or compromised data quality. A process was implemented to ensure that key staff based in The Gambia were trained to detect NTS ‘warning signs’ when collecting data so the UK based team could be immediately contacted.

These included the identification of (i) channels exhibiting unusually low raw intensity readings in reference to the values expected for the NTS system and (ii) channels showing oscillatory patterns inconsistent with the underlying physiology (i.e. heart rate or from other biological origin). The former was usually brought about by either an optode unclipping from the array, therefore affecting the readings in the channels defined by that optode and the neighbouring ones, whereas the latter was a consequence of eye tracker light being picked up by the fNIRS detectors. Staff were trained to address both of these issues by reattaching any loose optodes prior to the recording session and checking the fit of the headgear to ensure light from the eye tracker would not corrupt the signal (for further detail on this issue see section on ‘Simultaneous fNIRS/ET recording’ below). In addition to these checks, power level checks of all sources were performed in the beginning of the project to establish a baseline and then regularly repeated. Sources evidencing significant drops between such measurements were monitored and replaced at the next possible opportunity to prevent data loss.

Further, staff were trained to note signs of possible material damage to the headgear, such as stretchmarks or cracks in the silicone. Necessary spare parts were provided and stored on site, so that repairs could be carried out immediately via remote support from the system suppliers. This process has allowed local staff to detect, address and resolve all problems with the NTS fNIRS system to date, thus preventing delays and costly visits from specialists. This has been of particular importance, as in longitudinal, age-based design, adherence to timelines is essential to prevent missing data. Overall, none of the materials used had to be replaced more frequently than in previous studies, when accounting for the higher testing load.‘

Further, staff were trained to note signs of possible material damage to the headgear, such as stretchmarks or cracks in the silicone. Necessary spare parts were provided and stored on site, so that repairs could be carried out immediately via remote support from the system suppliers. This process has allowed local staff to detect, address and resolve all problems with the NTS fNIRS system to date, thus preventing delays and costly visits from specialists. This has been of particular importance, as in longitudinal, age-based design, adherence to timelines is essential to prevent missing data. Overall, none of the materials used had to be replaced more frequently than in previous studies, when accounting for the higher testing load.

***Transport.*** In contrast to other bulkier specialised research equipment, the instrumentation used in this project offers enough flexibility to be easily moved between different locations. As long as sufficient staff are trained to competency, it is possible for two people to dismantle, transport and reassemble our entire lab set up in less than 4 hours, ensuring minimal disruption to the infant testing schedule. Along with the maintenance process, the flexibility and portability offered by the fNIRS system allows it to function well in difficult working environments.

***Climate.*** The testing room at MRCG Keneba does not offer complete protection from the outside environment. While temperatures are high all year round, the contrasting seasonal patterns in The Gambia alternate between extreme dryness, creating a very dusty environment, and heavy rains leading to high humidity. Between testing sessions, the fNIRS system, fibres and headgear are covered appropriately for extra protection, and during the course of the longitudinal assessments, the instruments have not been found to be negatively affected by these conditions.

Due to the high temperatures, many of the infants were observed to sweat while wearing the fNIRS headgear, necessitating the use of air-conditioned rooms for testing. During pilot studies, high temperatures and sweating were found to increase the risk of movement of the headgear relative to the infant’s head resulting in misalignment of the channels and increased noise in the data, an issue that could be fully addressed by conducting all studies in air-conditioned rooms.

**Figure 3.**
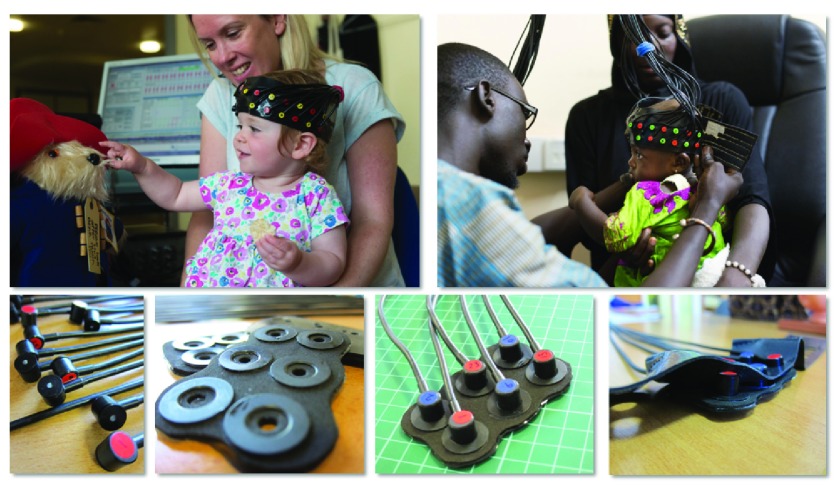
Illustration of fNIRS headgear used at BRIGHT study sites. Upper panel: fNIRS bespoke headgear worn by infants in the BRIGHT project in the UK and The Gambia. Lower panel: optodes and fibres (far left), sensor arrays with clip-on optode holders (left), source (red) and detector (blue) fibres clipped into array (right) and headband and optode array combined (far right). Image copyright: Ian Farrell (top panel), Sarah Lloyd-Fox (bottom panel).

***Headgear.*** The headgear used for the fNIRS measures, which has been designed by researchers at Birkbeck and UCL ( Lloyd-Fox *et al.*, 2010), has been highly successful in previous research projects and been purchased for use in eight other research labs. However, while this bespoke headgear has many strengths, including flexibility, secure fit on head and easy usability with infants, it was originally designed for smaller-scale projects of limited duration. During the BRIGHT project over a period of 24 testing months the Gambian fNIRS system and headgear have been used with over 600 infants. The frequency of use and the high ambient temperatures, has necessitated the replacement of some of the silicone parts of the headgear more frequently than in previous studies. Local staff have been trained to repair headgear on site, minimising data loss. Any damage and/or required modifications to the headgear are recorded in a shared document between the Gambian and UK sites to enable teams to anticipate and be ready for any issues. We have further established a process in which plastic and silicone components that were originally handmade can now be 3D printed, greatly reducing the time necessary for production and repairs *.* Figure 3 illustrates the headgear used in the BRIGHT project.

Previous studies of infant growth ( Prentice *et al*., 2013), provided head circumference measures which enabled us to design a range of headgear of different sizes. This ensured that optodes were consistently placed in accordance to anatomical landmarks of the infants’ heads as they grew. Due to the higher variance of head circumferences in Gambian compared to UK infants, the choice of headgear has been guided by individual infants’ head size, rather than the age point at which they are assessed. As frequent switches of the headgear can weaken some of its components (i.e. silicone headbands, clip-in optode holders and silicone layers within the sensor arrays), some steps have been taken to ensure successful data collection despite this issue. Firstly, 3D printed versions of the clip-in optode holders were developed, to replace the handcrafted versions, making them more durable, easier to switch and faster to replicate whenever spares are needed. Secondly, research staff received extensive training in sensitive handling of the headgear and NIRS system including; frequent trips by UK fNIRS expert researchers, training videos for all steps of fNIRS headgear and system maintenance which were shared across sites and weekly group meetings during which any issues could be discussed.

**Figure 4.**
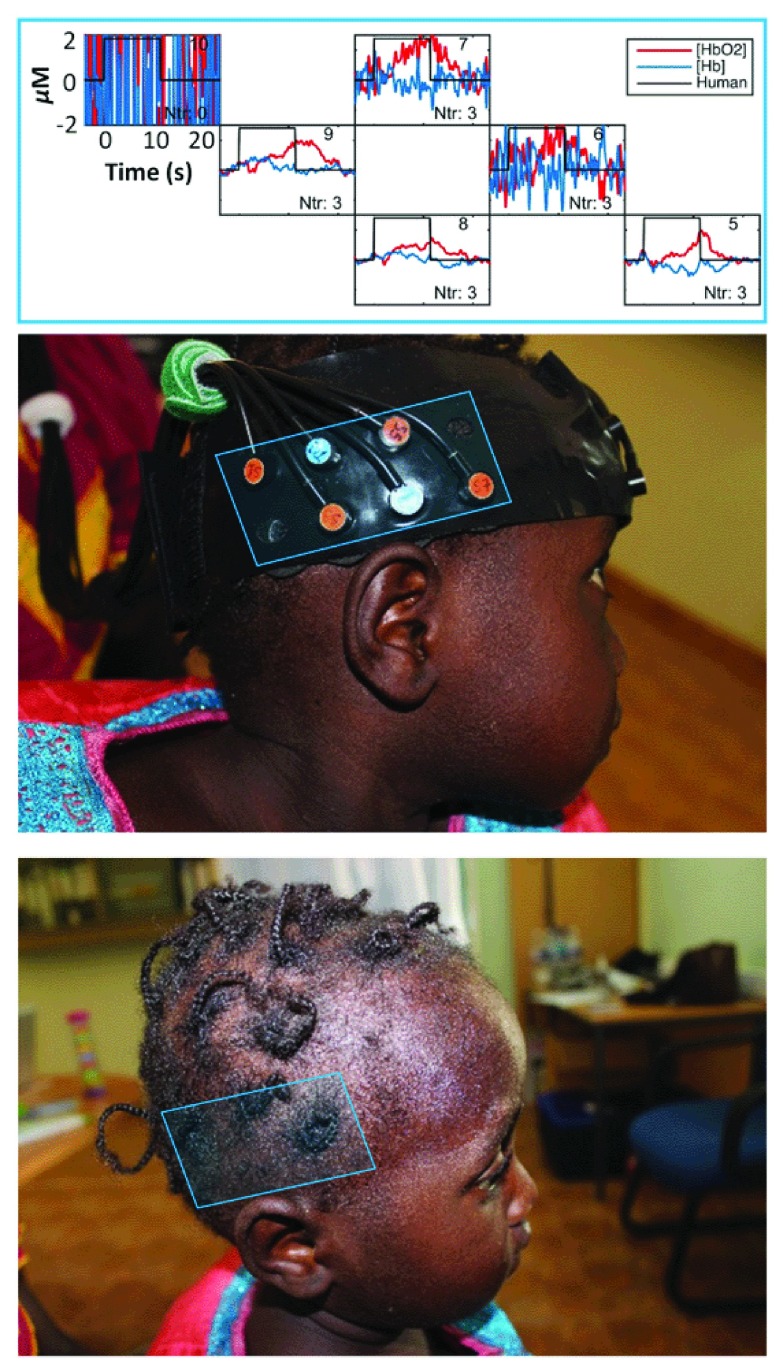
Effects of hair braids on fNIRS signal quality in posterior temporal and frontal regions. Plots show changes in the haemodynamic response of HbO _2_ (red) and HHb (blue) at each channel position on the child’s head. A close correspondence can be seen between the location of hair braids and noise levels in the signal. It can also be seen that it is possible to obtain good signal to noise in channels neighbouring those affected by the braiding. Image copyright: Sarah Lloyd-Fox.

***Dark skin and hair.*** One issue we anticipated was the increased light attenuation in dark-skinned subjects with frequently braided hair. Dark skin did not affect the signal to noise on our data during the initial pilot phase of the project ( Lloyd-Fox *et al.*, 2014). The power of the light sources for the NTS optical imaging system we are using is 3.0 mW for the 780 nm and 4.5 mW for the 850 nm, both of which adhere to the laser safety standards for these systems ( Everdell *et al.*, 2005). In The Gambia, from around one year of age, many of the girls start to have their hair tied or braided which creates visible artefacts in the data ( Figure 4). Where this is an issue, we ask mothers to undo the braids and ties for the duration of the fNIRS assessment. Whenever was found to be found to be impossible, were still able to collect data from surrounding channels, which were not affected by hair to measure responses in some of the neighbouring regions of interest. *The specific criteria varied between paradigms, according to hypotheses regarding localisation and lateralisation of the responses. Wherever studies were replications of previous studies in the UK, the same criteria for inclusion and exclusion of datasets were applied. Between the two parallel sites in the UK and The Gambia, the same criteria were applied, and no higher leniency had to be applied at the Gambian site. One important consideration in this context is that even though darker skin and hair can increase the noise of a measurement, this influence is constant across the entirety of the session and does not differ between baseline and task phase, or between conditions. As fNIRS measures relative changes in oxy-and deoxygenated haemoglobin changes, these relative measures are uniformly affected under these conditions. Similarly, as infants were assessed longitudinally, age-related changes were less affected by these issues.*

**Figure 5.**
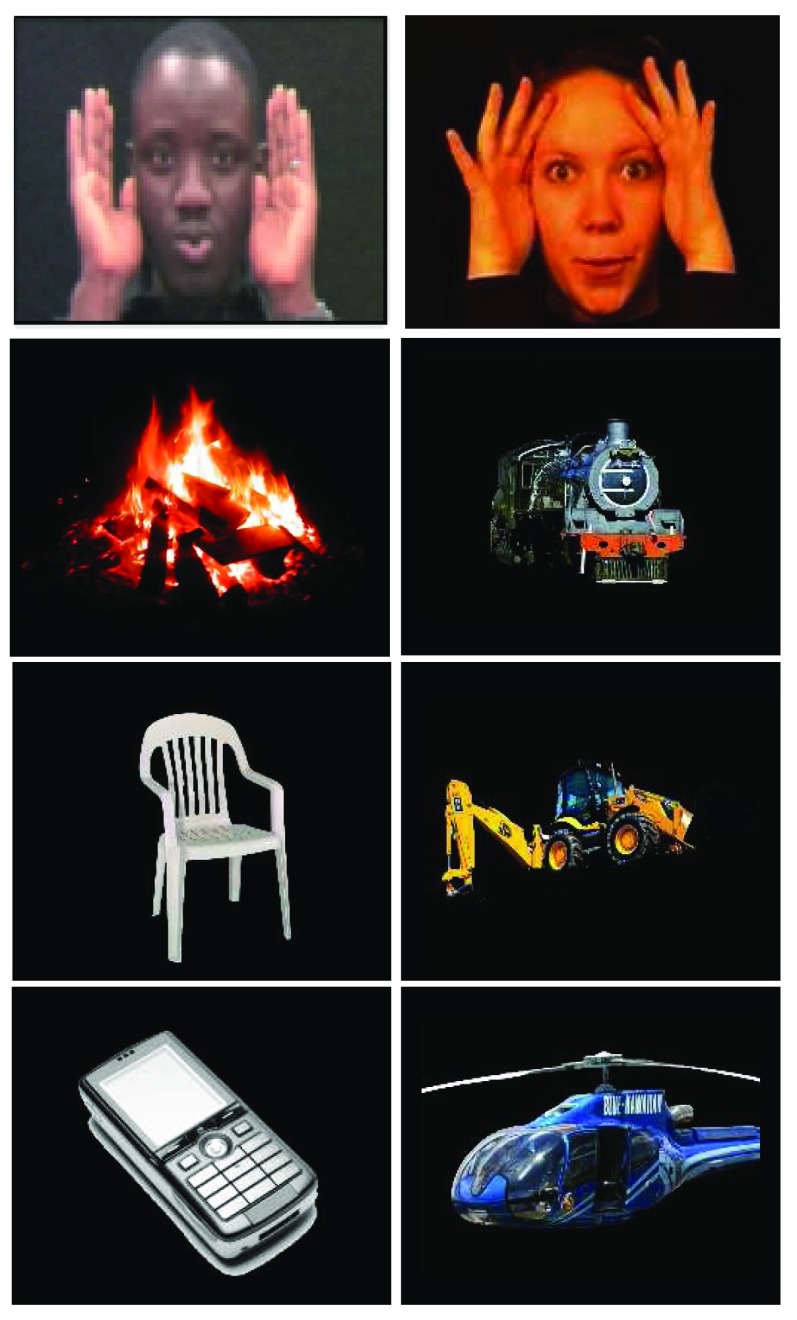
Stimuli used in fNIRS social vs. non-social paradigm used in The Gambia (left) and the UK (right). Top row shows examples of social stimuli used at either site. Bottom rows show examples of non-social still images specific to cultural contexts.

***Stimuli.*** While the same study paradigms are used for both the Gambian and UK sites, any stimuli containing actors or spoken language needed to be adapted for use with Gambian infants. One paradigm employed in the BRIGHT project has already been extensively used in previous studies (i.e. Braukmann *et al.*, 2018; Lloyd-Fox *et al.*, 2009; Lloyd-Fox *et al.*, 2018) and re-filmed for use in The Gambia ( Lloyd-Fox *et al.*, 2014). Videos used in this social vs. non- social paradigm which display different actors performing gesture games (e.g. ‘peek-a-boo’, ‘incy-wincy spider’) were re-filmed using Gambian actors whilst being careful to retain the required stimulus length, timing of gestures and facial expressions ( Figure 5). The still images forming the baseline of the paradigm were adjusted to show everyday objects found in the village and the clinic at MRCG Keneba. Pictures were taken during an initial visit to MRCG Keneba and subsequently edited to visually match the still images used in the UK in terms of background and visual space covered relative to the screen. While in the established UK version of the paradigm objects formed a coherent category (‘objects of transport’), it was not possible to find a sufficient range of objects from any one category in The Gambia, so a range of objects from different categories was chosen ( Figure 5). These objects included a chair, a well, a mosque, a radio, a phone and a fire place, which all infants were expected to be familiar with from within their village and home environment. In the UK version, even though we do not expect all of the infants to have seen all of the transport objects used in the paradigm, we assumed that they had been exposed to representations of the objects in books or videos. As infants recruited at the Gambian site of this project do not regularly have access to books or television, we placed an emphasis on the use of objects which are present in their day-to-day lives. Following this successful adaptation for the Gambian context, these stimuli were also refilmed for implementation of this paradigm in Bangladesh (as part of The Bangladesh Early Adversity Neuroimaging (BEAN) Project).

Similar steps were followed for the other fNIRS paradigms. In the working memory paradigm infants are presented with a video in which an actor hides an object inside a box for varying lengths of time. Videos were filmed using the same objects and using the exact same timings across sites to avoid confounds with the main experimental manipulation. The functional connectivity paradigm consists of videos showing actors reciting nursery rhymes. Culturally appropriate nursery rhymes were used at both sites to present infants with familiar social input. To generate these paradigms videos of both the English- and Mandinka-speaking actors were filmed in the same location at the same time with volunteers from The Gambia who were visiting the UK at the time of filming.

For the HaND task the text of the auditory stimuli was first translated from English to the local language of Mandinka (with a team of Mandinka speaking researchers from within the BRIGHT project), then retranslated to English (by a different Mandinka speaking researcher, not part of the BRIGHT project) to ensure the meaning had not changed. Mandinka is not a formal written language and so these steps were taken to ensure accurate translation (see Lloyd-Fox *et al.*, 2019). As several languages are spoken in The Gambia and all stimuli and questionnaires needed to be transferred into participants’ native language—with the majority of families requiring interview style questions due to illiteracy—it was decided to limit our recruitment to the Mandinka ethnic group, which is the prevailing culture and language in the West Kiang region of The Gambia where our project is based.

### EEG

EEG is a widely used technique in infant research, and as such has considerably improved our understanding of early neurocognitive development over the past decades ( de Haan, 2013; deBoer *et al.*, 2005). EEG recordings can be regarded as a transcription of electrophysiological activity, originating primarily in the brain. Synchronous electrical activation generated by large populations of neurons in cortical brain regions can be measured by electrodes placed on the surface of the scalp ( de Haan, 2013). An EEG primarily reflects activity from cortical pyramidal cells. These cells have a high density within the cortex and are aligned perpendicular to the surface of the scalp. As these cells are comparably large and uniformly aligned, their activity becomes strong enough to be read out by electrodes, placed on the head on scalp level.

**Figure 6.**
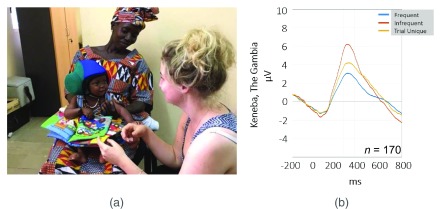
Set up and output of BRIGHT EEG study. ( **a**) Set up of EEG assessment at 5 months at MRCG Keneba. Infant is seated on parent’s lap and silently interacts with one of the researchers. The wireless EEG system and headphones allow for some flexibility regarding the infant’s position in the room. ( **b**) Grand Average of ERP response of n = 170 one-month-old infants tested at MRCG Keneba for frequent tones (blue), infrequent sounds (red) and trial unique sounds (yellow) showing differential neural responses across these sound categories. Image copyright: Laura Katus.

EEG signals can be analysed in a number of ways, one commonly used approach lies in the examination of event-related potentials (ERPs). To generate ERPs, the continuous EEG recording is subdivided into event-related epochs, changes during which can be used to examine rapid changes in electrical activity generated in the brain across repeated stimuli. By averaging tightly time-locked responses across trials, noise can be reduced and changes that consistently occur in response to a stimulus can be isolated. Figure 6b shows the ERP response measured in a group of 1-month-old Gambian infants as part of the BRIGHT project. The positive and negative deflections in the ERP waveform can be analysed in terms of their magnitude and latency of occurrence, which can then be compared across experimental conditions, age points or study cohorts.

The majority of developmental EEG and ERP research has been conducted in high-income countries. Despite previous successful implementations of this technique in the study of child development in sub- Saharan Africa ( Kihara *et al*., 2010), there remains a scarcity of literature using these methods to study infant neurodevelopment in low- and middle-income settings.

The ERP paradigm implemented as part of the BRIGHT project assesses how rapidly infants habituate to recurrent familiar stimuli and detect novel auditory stimuli. The ERP observed in this task as well as its changes across development have been well characterised across infant development ( Kushnerenko *et al*., 2013). During the data collection, infants listen to the auditory stimuli through wireless headphones while one of the researchers quietly interacts with them ( Figure 6a). The wireless system used in this project allows us to collect data while the infant is sitting on their parent’s lap, lying on a mat on the floor or being held or carried around the room during the study, if necessary. Data retention of 80% at one month and 70% at the five-month age point is slightly higher compared to previous research in these age groups.

***Transport.*** To facilitate transport of the EEG equipment, data was collected using a wireless, Neurolectrics Enobio8 system. All equipment needed to run this EEG study is battery powered and can easily fit into a small bag. The connection between the amplifier and acquisition laptop is established locally via Bluetooth and can thus easily be made to work in the field.

***Climate.*** Similar to the fNIRS data, EEG data quality is negatively affected by high temperatures as sweating can mask and distort the measured signal. Best data quality is thus achieved in sufficiently air-conditioned environments. Other projects have reported that EEG electrodes can be negatively affected by humidity, leading to reduced data quality ( Kappenman & Luck, 2010); however, this issue has not been encountered with the recording system used in this project. 

***Electrical noise.*** EEG data is negatively affected by electrical line noise, which is produced by stray electromagnetic signals in proximity to the system. In the absence of testing rooms designed specifically to shield against this, pilot studies were conducted to determine the extent of the problem in the local environment in both the UK and The Gambia. At MRCG Keneba, the air conditioning was found to cause some interference when switched on, therefore its use was limited to cool the room down prior to the EEG studies.

***Stimuli.*** The EEG study implemented in the BRIGHT project consists of pure tones, and a range of deviant sounds (adapted from Kushnerenko *et al.*, 2007). As these sounds hold little inherent cultural or social information, these stimuli could easily be used at both testing sites and at various age points throughout the project.

### Eye tracking

**Figure 7.**
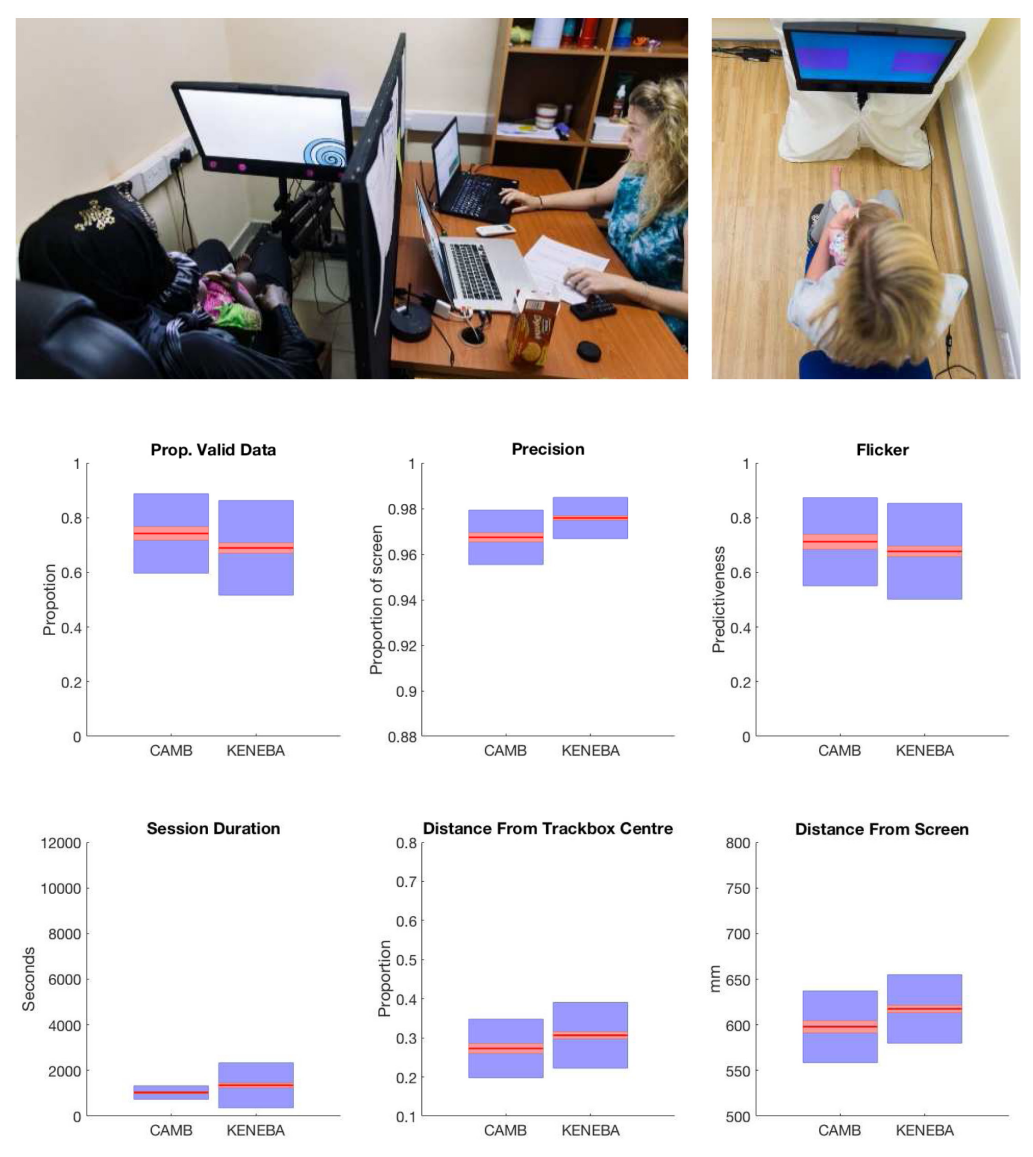
Set up and data quality metrics of BRIGHT ET study. Upper panels: BRIGHT project eye tracker setup, with infant seated on parent’s lap while attending to the screen. Eye tracking is integrated in a panel just below the screen (upper left panel). Lower panels: six measures of data quality. Clockwise from top left, ( **a**) proportion of samples in which the eyes were detected; ( **b**) spatial precision; ( **c**) flicker, the ratio of validity for sample n to sample n+1; ( **d**) the duration of each eye tracking session; ( **e**) distance of the infant from the centre of the "track box", the volume in which the eye tracker can detect eyes (the centre of which yields optimal data quality); ( **e**) the distance of the infant from the screen. Red lines represent the sample mean, red boxes represent 95% confidence intervals, blue boxes represent 1 SD.

Eye tracking relies on the acquisition of high-speed (up to 500 Hz) digital infrared pictures of the participant’s eyes, and software algorithms identify corneal and retinal reflections. From these, the rotation of the eye in the x and y axes is estimated. A brief (~20 s) calibration procedure run at the start of each assessment relates angular rotations of the eye to known spatial positions on a screen. From here, the eye tracker records the gaze coordinates on each sample of data, as well as pupil size and the three-dimensional location of each eye. The BRIGHT project uses a Tobii TX-300 (Tobii AB, Sweden) remote eye tracker, with an integrated screen on which stimuli were presented ( Figure 7). Infants were assessed while seated on their parent’s lap, between plain-coloured dividers to reduce distractions ( Figure 7).

Our experience in eye tracking with infants is that there is a trade-off between sampling rate and data quality (lost samples, accuracy and precision). For this reason we set the sampling rate of the eye trackers to 60Hz. This reduces the temporal precision of each sample of data to 16ms (vs 8ms at 120hz or 4ms at 300Hz). Our understanding of the mechanism behind this trade-off is that faster sampling rates mean less light hitting the eye tracker’s sensor on each sample, leading to a poorer quality eye image. Given that we could not exercise complete control over ambient lighting levels, particularly in the Gambia, we decided that it was worth suffering the loss of temporal resolution in order to a) make the process of calibrating and tracking the infants as easy as possible for non-eye tracking experts; and b) to maximise data quality, thus minimising dropout from analysis and longitudinal retention.

***Task battery.*** A number of tasks are presented, measuring a wide variety of cognitive domains, such as cognitive control and reversal learning, attention shifting (gap/overlap), habituation to social and non-social stimuli, visual search, contingency preference, and a selection of static and dynamic stimuli. The majority of the tasks used in the BRIGHT project were either in use, or already validated, in associated studies, such as The British Autism Study of Infant Siblings (BASIS), European Autism Interventions (EU-AIMS,), the Autism Biomarker Consortium for Clinical Trials (ABC-CT) and the Developing Human Connectome Project (DHCP). 

Given the age of the infants, all tasks are passively viewed, and to minimise habituation to a particular task, or to the screen, short blocks of each task are interspersed with each other throughout the 20-minute battery. 

***Data quality.*** Data quality can affect findings in any assessment but is particularly important in eye tracking. Missing data can give rise to artificially long or short reaction time estimates, while spatial error can result in miscalculated looking time durations. Sources of differential data quality across ages are broadly of three categories:

1) Infant behaviour: looking away, blinking, moving within reach of the eye tracker (which increases spatial error) or moving outside of the trackable zone (which leads to missing data).
2) Physiological characteristics: the relative moistness of the eyes of young infants can lead to extra corneal reflections that may confuse the software algorithms that the eye tracker uses. Eye colour can lead to differences in data quality and varies across ethnicities.
3) Experimenter factors: experimenters receive constant feedback on gaze quality during an assessment and can make the decision to reposition an infant in relation to the eye tracker, take a break, trigger an attention-getter or give a snack. Differences in how or when these decisions are taken based on individual infant response can lead to differential data quality between sites.

In order to provide rapid feedback on these causal factors behind varying data quality, automated quality control procedures are being used. These summarise each session, and each trial of each experimental task, for temporal and spatial error, missing data, and trial validity ( Figure 7). Assessing the quality of data in this way also allows one to address any residual differences statistically in the analysis stage.

A final aspect of the environment that affects data quality of eye tracking is the ambient lighting. In ideal conditions, daylight (which contains some frequencies in the infrared spectrum) would be eliminated from testing rooms. Artificial light would be diffused, to avoid spot reflections on the pupil that may confuse the eye tracker’s algorithm, and the intensity of light would be maintained at ~300 lux. It was not possible to implement these conditions in MRCG Keneba, so instead the eye tracking studies are being conducted in a windowless room with one overhead light. Despite this difference, and those described above, data quality is comparable at each site. Task-related measures of data quality, such as spatial error, are very similar across sites as well.

## Feasibility of cross-modal assessment in a large-scale longitudinal project

### Differences across ages and sites

Many tasks implemented in the current project, and neurodevelopmental studies generally, rely on fast paced, or repetitive audio-visual stimulus presentation. Especially at younger age points, a large proportion of infants required prolonged resting periods in between these assessments to ensure data quality for subsequent measures was not compromised. To avoid data loss, we frequently operate a testing schedule where multiple infants are invited and can be assessed with the different measures in different rooms when they are able to. This infant-led approach was also applied in adjusting protocols per age point so that tasks that the majority of infants would not engage in could be omitted from the protocol to ensure data retention of subsequent measures.

### Simultaneous fNIRS/ET recording

**Figure 8.**
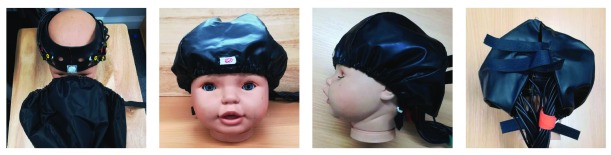
Headcap used to shield fNIRS headband from near infrared light. The cap is attached to the fNIRS headgear at the infant’s forehead using Velcro. The lower edge of the headband has elastic spanning the lower rim of the fNIRS cap. Velcro strips in the back of the headband allow quick adjustment for different head sizes to prevent delays in capping.

 Through simultaneous acquisition of fNIRS and ET data we were able to establish an automated evaluation of infants’ looking behaviour in relation to the fNIRS stimuli, facilitating checks of infant attention during data analysis. Initially, high interference was caused in the fNIRS recording due to the simultaneous use of the eye tracker, as it emits near-infrared light at a wavelength of 850 nm which lies within the sensitive range of the fNIRS detectors. To address this, a specifically designed headgear cover was used to ensure that the NIR light from the ET system did not reach the fNIRS detectors. Good results (high signal to noise in the measured data) were achieved using a design consisting of two layers of blackout rubberized fabric (BK5, Thorlabs, Inc.), assembled as a headcap ( Figure 8) which completely covers the fNIRS headgear and is attached to it at the infant’s forehead using Velcro. The lower edge of the headband has elastic spanning the lower rim of the fNIRS cap, extending down over the ears on either side. Velcro strips in the back of the hat make it easily adjustable for a range of head sizes, and easy to be placed correctly and quickly so that infants do not become fidgety while the caps are placed. Following training to ensure correct placement, the cap blocks out the majority of the ET light which enables us to simultaneously record high-quality data from both modalities.

### Acceptance of methodologies by population

 We worked in close contact with the local community to ensure all measures within the BRIGHT project would be well accepted by parents as well as the wider community. Wherever possible, staff that had already been involved in testing since the initial piloting phase were employed. Their support was vital in communicating our aims and explaining the methods involved to participating families. This has resulted in us meeting our recruitment targets, and to date, retaining a high proportion of participants at each age point. 

### Staff training

The BRIGHT project in The Gambia is run by a largely Gambian field staff with extensive experience in organising study visits and with good rapport with the local community from whom participants were recruited. Prior to working on the BRIGHT project, none of the core testing team was experienced with neuropsychological assessments or neuroimaging. Due to the relatively minimal training that is required to administer fNIRS, EEG and ET, and highly motivated staff members, it was possible to train staff to independently collect high-quality data on all assessments. In order to maximise data quality and reduce the training burden on each team member, an approach was used in which different members of the team were trained as experts for certain assessments, while still being sufficiently familiar with all other methods to support data collection across the project. Prior to each of the longitudinal time points, assessments were piloted and practiced by the team in the UK, before conducting targeted training and piloting at the MRCG Keneba field station with Gambia-based staff.

This helped to detect any issues early on and to ensure the standardisation of assessments across sites. It has been necessary to continuously monitor data quality and offer additional training and advice during regular discussions between staff at both testing sites.

### Seasonality and religious observances

 As infants are assessed throughout the entire year, we had to take into account that during harvest season mothers carry out work in the fields and are therefore less available to come in for study visits. Further, religious observances such as Ramadan had to be considered, during which visits needed to be scheduled soon after sunrise so that fasting mothers and staff as well as breast feeding infants were not too tired to complete the assessments.

### Challenges of different age groups

The longitudinal design of the project necessitated some adjustments to accommodate the specific needs of each age group. For assessments at the one-month age point infants were assessed asleep, and thus required a quiet testing environment despite many other ongoing projects in close proximity of the lab. At the five-month age point it had to be ensured that the optical fibres of the fNIRS headgear were light enough for infants to wear despite limited neck strength, especially taking into consideration the typically smaller body size of infants recruited in The Gambia.

At the eight-month and subsequent age points, as expected from previous research, infant’s ability and motivation to reach for, and pull on, the fNIRS and EEG headgear increased, making it necessary to occupy their hands during studies by having them hold a small toy or a rice cake. The majority of infants in the West Kiang region of The Gambia, are not frequently exposed to Caucasian people. It was observed that, from around 12 months onwards, some Gambian infants started to react fearfully to the Caucasian staff in the testing team, meaning that in those instances testing would be completed by only Gambian staff to ensure infants were able to engage with all assessments.

### Transfer of large data volumes

The sample recruited in The Gambia and the number of assessments performed per time point are considerably larger than in most other neurodevelopmental projects. Several of the measures involve the collection of large data files (e.g. video recordings of infant behaviour during studies, pictures documenting the fit of fNIRS and EEG headgear) which require transfer to the UK based analysis centres for checks of data integrity and quality. The network connection at MRCG Keneba is often not able to support transfer of large data volumes and can also be negatively affected by the strong seasonal rains.

To counter this, a three-step protocol for data transfer was implemented consisting of initial local backups at each site. These are organised by task (fNIRS, EEG, eye tracking, behavioural), modality (imaging data, video recordings, Matlab output, observation sheets, pictures etc.) and age point, in pre-defined folders that mirror the final storage folder structure. The data was then transferred to an SFTP server, which acts as an intermediate storage unit, by using a synchronisation option to ensure all newly acquired data are transferred. Finally, these intermediate folders are synchronised with those stored on the final server, by a similar synchronisation process. As it cannot be guaranteed that data can be transferred immediately after testing the local backup protocol specifies for two copies of each files to be saved independently. Software updates which necessitate large data files, are transported on physical hard drives by staff travelling between sites. 

### Harmonised stimulus presentation

 In establishing a protocol with such a rich range of stimuli across a number of assessment modalities, it was crucial to ensure standardisation of stimuli presented at each site and each age point, whilst maintaining the flexibility to alter the protocol if certain aspects of it were not working in practice.

This was particularly important for the study arm located at MRCG Keneba, where infants were not used to watching a screen, and where there were no past studies to refer to when assessing the suitability of, and infant preferences for, particular stimuli. To ensure flexibility in altering task presentation where necessary without requiring large amounts of data to be transferred between sites, software specifically for multisite longitudinal studies was used. The Task Engine stimulus presentation and data acquisition software was developed at Birkbeck, University of London ( https://sites.google.com/site/taskenginedoc). By automating many of the technical aspects of running the various studies, the Task Engine also enables data collection to be independently undertaken by non-expert staff members.

In addition to multisite stimulus presentation, the Task Engine framework also ensures temporal synchronisation of multiple stimulus modalities. This enabled eye tracking data to be concurrently acquired during our fNIRS battery. In order to ensure that brain responses were only measured for stimuli that infants were actually looking at, we used this eye tracking data to facilitate attention coding of the fNIRS tasks. Periods where the eye tracker detected the eyes and when the infant was attending were automatically marked as valid. Periods where the eye tracker did not report that the infant attending were manually reviewed to determine whether 1) the infant was indeed not attending to the screen; or 2) the eye tracker had failed to detect the infant’s eyes. This process significantly improved the rate at which attention coding was performed.

## Ethical approval and consent

Ethical approval was obtained for each study site separately. In The Gambia, the BRIGHT project was approved by the local SCC (project title ‘Developing brain function for age curves from birth using novel biomarkers of neurocognitive function’, SCC number 1451v2, on 13-01-2016). In the UK, ethical approval was granted by the NHS Health Research Authority (project title ‘Developing brain function for age curves from birth using novel biomarkers of neurocognitive function.’, reference 15/EE/0202, project 178682 on 03-08-2015). 

Consent was obtained from all participating families for photographs taken of themselves and their infants during assessments and for their use in scientific publications and manuscripts.

Consent was also obtained from any of the BRIGHT researchers present with the families in these photos.

## Conclusion

We have successfully introduced an extensive longitudinal protocol assessing neurocognitive development in young infants in rural Gambia, alongside a parallel study in the UK. In particular, the unprecedented implementation of fNIRS, EEG and ET in a single protocol in a low-income setting is encouraging and highlights the potential of these methods for use in similar locales around the world.

Indeed, a subset of the fNIRS tasks included in this protocol has already been taken up and are currently being implemented in The BEAN Project in Dhaka, Bangladesh, in the study of infectious disease and nutrition in infants and children ( Storrs, 2017) and in Uttar Pradesh, India, in the context of exposure of environmental risk factors (for a description of this project in context of another fNIRS paradigm see Wijeakumar *et al*., 2019).

Implementing unified protocols and analysis streams across projects will enable comparative conclusions on the impact of environmental adversity encountered in different settings. It will further aid the identification of early markers specific to and common across the different study sites. The definition of these markers will be the starting point towards identifying factors to be targeted in future interventions. To further the utility of the proposed methods for clinical and diagnostic purposes, current advances in the development of battery-powered wearable equipment hold potential of enabling routine assessment of infants on an even larger scale. This will further enable us to detect at-risk infants and intervene at an early stage, thus lessening the impact of environmental risk factors before the infant is exposed to them for prolonged periods of time, which will have a lasting positive effect on their developmental outcome.

## Data availability

All data underlying the results are available as part of the article and no additional source data are required.
