# Supplementary material for: Implementing neuroimaging and eye tracking methods to assess neurocognitive development of young infants in low- and middle-income countries
Source: Gates Open Res. 2019 Aug 27;3:1113. Originally published 2019 Apr 5. [Version 2] doi: 10.12688/gatesopenres.12951.2 (PMC6719506; doi:10.12688/gatesopenres.12951.2)
Supplement: Peer review history

# Peer review: Reviewer response for version 2

**DOI:** 10.21956/gatesopenres.14174.r27735

**Published:** 2019-09-02

**Authors:** John P. Spencer, Samuel H. Forbes

**Affiliations:** 1 School of Psychology, Lawrence Stenhouse Building, University of East Anglia (UEA), Norwich, UK; 2 School of Psychology, Lawrence Stenhouse Building, University of East Anglia (UEA), Norwich, UK

I am satisfied that the authors have addressed my concerns.

We confirm that we have read this submission and believe that we have an appropriate level of expertise to confirm that it is of an acceptable scientific standard.

# Peer review: Reviewer response for version 2

**DOI:** 10.21956/gatesopenres.14174.r27734

**Published:** 2019-08-29

**Authors:** Wanze Xie

**Affiliations:** 1 Boston Children's Hospital, Boston, MA, USA; 2 Harvard Medical School, Boston, MA, USA

The authors have addressed all my questions, and thus I have no further comments to make.

I confirm that I have read this submission and believe that I have an appropriate level of expertise to confirm that it is of an acceptable scientific standard.

# Peer review: Reviewer response for version 1

**DOI:** 10.21956/gatesopenres.14056.r27083

**Published:** 2019-05-17

**Authors:** Wanze Xie

**Affiliations:** 1 Boston Children's Hospital, Boston, MA, USA; 2 Harvard Medical School, Boston, MA, USA

This paper introduces the usage of neuroimaging tools and behavioral measures to investigate neurocognitive development in children living in low-income countries. The authors discussed the implementation of a combination of measures (e.g., EEG, fNIRS and eye-tracking) in rural Gambia, West Africa in the Brain Imaging for Global Health (BRIGHT) project. Overall, this paper is well-written and provides a comprehensive reference or guidance to other projects aimed to study brain and cognitive development among children living in low-resource settings, and thus has the potential to advance the field further. In my opinion, there are only a few minor issues requiring revision or clarification, which are listed in the order of appearance in the paper. 

1. Introduction, 3 ^rd ^paragraph: I would recommend the authors to focus on discussing how neuroimaging tools are capable to fill gaps and add to the existing findings obtained from behavioral assessments, rather than listing the “limitations” of the behavioral assessments for a couple of reasons. First, the entire field has learned a lot by using those behavioral assessments/measures and most of the ongoing projects on global health are still using all kinds of behavioral assessments to obtain critical and unique information on child development. In the meantime, behavioral assessments do have advantages over brain measures, such as the relatively higher reliability and possibility to reproduce the findings. 
  Second, some of the statements about the “limitations” of the behavioral assessments may be inaccurate, and they also exist in brain measures. For instance, “they require extensive training to ensure inter-observer standardization.” This happens to brain and other behavioral measures as well. 
  To sum up, I get the authors’ points, but I do think a couple of sentences in the paragraph should be rephrased. 
2. As the authors may have already noticed, there are a few other ongoing projects that also implemented similar neuroimaging tools to study neurocognitive development in low-income countries. For example, projects led by other researchers (e.g., Drs., John Spencer, Charles A. Nelson, Elizabeth Prado, Shekhar Saxena, etc.) have already published studies using the same kind of tools implemented in the BRIGHT project in low- and middle-income countries. Given the title of the paper is “Implementing … in low- and middle-income countries” not *just* in Gambia, I think a brief overview of a few other projects in the introduction before going into the details of the BRIGHT project can be informative to the readers, as it would indicate the importance of studying neurocognitive development in the context of global health and the efforts that people have put into this area. This could be then followed by the description of the uniqueness of the BRIGHT project.  
3. “Novel methods implemented in BRIGHT project”: Again, these are not novel methods. Maybe change it to “Neuroimaging methods …”? 
4. fNIRS section – *Dark skin and hair*: It’s interesting to know that the color of our skin and our hair have an impact on fNIRS signals. I wonder if the authors have ever tested this with empirical data. How different could the results be between participants with different skin colors, i.e., how big could the effect be? This could be an important question for other projects targeted on different populations.  
5. EEG section: The authors claimed that “the most commonly used approach lies in the examination of ERPs”. I don’t know if people will all agree with this statement. Apparently, ERP is one of the most commonly utilized EEG approaches, but I am not sure if it is fine to say it is the most.
  In the same section, “The vast majority of developmental EEG and ERP research … Western …” I am not sure this is accurate either: there are other places in the world other than Western and African countries, e.g., a large number of EEG/ERP studies have been conducted and published with children in East Asia. Maybe rephrase it as “There is scarcity of … in the literature regarding … in Africa or low-income countries”? 

I confirm that I have read this submission and believe that I have an appropriate level of expertise to confirm that it is of an acceptable scientific standard.

# Response

**Published:** 2019-08-01

**Authors:** Laura Katus

**Affiliations:** University College London, UK

“1. Introduction, 3rd paragraph: I would recommend the authors to focus on discussing how neuroimaging tools are capable to fill gaps and add to the existing findings obtained from behavioral assessments, rather than listing the “limitations” of the behavioral assessments for a couple of reasons. First, the entire field has learned a lot by using those behavioral assessments/measures and most of the ongoing projects on global health are still using all kinds of behavioral assessments to obtain critical and unique information on child development. In the meantime, behavioral assessments do have advantages over brain measures, such as the relatively higher reliability and possibility to reproduce the findings. Second, some of the statements about the “limitations” of the behavioral assessments may be inaccurate, and they also exist in brain measures. For instance, “they require extensive training to ensure inter-observer standardization.” This happens to brain and other behavioral measures as well.

To sum up, I get the authors’ points, but I do think a couple of sentences in the paragraph should be rephrased.”

We appreciate the suggestion and have changed the emphasis of this section accordingly. We have indicated the relative strengths of both assessments to highlight when the methods described in the current paper might be of greatest value.

*‘One limiting factor in studying neurodevelopmental trajectories in LMICs is the limited availability of assessment tools that can offer objective, mechanistic insight into brain development, with proven validity across cultures. Much neurodevelopmental research to date has been based on behavioural assessments. While these assessments have vastly advanced this field of research, they have some limitations when used in longitudinal, multicentre cross-cultural research. When used during the first year or life, the predictive validity of behavioural assessments has been found to be reduced, especially when used to assess at-risk populations, such as infants with very low birthweight ( Hack et al., 2005) and infants with familial risk of autism ( Elsabbagh & Johnson, 2010; Yirmiya & Charman, 2010). While behavioural responses sufficient to indicate altered developmental pathways are often only evident towards the second year of life, significantly earlier changes in brain functional and anatomical specialisation have been found in the first months of life in at-risk populations ( Bishop, 2007; Elsabbagh et al., 2012; Lloyd-Fox et al., 2017). This indicates the potential for an earlier window of detection and implementation of intervention than behavioural measures commonly allow. When implemented after infants first birthday, neurobehavioural assessments become much more accurate and reproducible, but also time consuming, limiting their potential for routine use in clinical practice. While behavioural assessments have and continue to offer valuable insights in neurodevelopmental research, neuroimaging and eye tracking can provide additional insights; implementing these during the first months of life holds potential to uncover early correlates of later behavioural outcome, and importantly can inform our understanding of underlying mechanisms in the developing brain that might give rise to either typical or atypical outcomes.’*

“2. As the authors may have already noticed, there are a few other ongoing projects that also implemented similar neuroimaging tools to study neurocognitive development in low-income countries. For example, projects led by other researchers (e.g., Drs., John Spencer, Charles A. Nelson, Elizabeth Prado, Shekhar Saxena, etc.) have already published studies using the same kind of tools implemented in the BRIGHT project in low- and middle-income countries. Given the title of the paper is “Implementing ... in low- and middle-income countries” not *just *in Gambia, I think a brief overview of a few other projects in the introduction before going into the details of the BRIGHT project can be informative to the readers, as it would indicate the importance of studying neurocognitive development in the context of global health and the efforts that people have put into this area. This could be then followed by the description of the uniqueness of the BRIGHT project.”

We appreciate this suggestion and we are glad to be able to include the most recent publications from the relevant groups. While the work of the groups of Drs Charles Nelson and John Spencer were referenced in the discussion, we have now moved the mention of these projects to the introduction of the manuscript. We have made reference to the latest publications by Charles Nelson’s and John Spencer’s group as below. Despite our awareness that the work of other groups (i.e. Elizabeth Prado’s and Shekhar Saxena’s) is highly relevant in this context, to our knowledge they have not yet published any empirical data from these investigations, which is why we do not reference their work explicitly here.

*‘fNIRS, EEG and ET have been implemented in high-income countries for many years, and several groups have now begun to implement these methods in populations in LMICs, including Bangladesh (Jensen et al., 2019, Perdue et al., 2019, Xie et al., 2018) Brazil (Shephard et al., 2019) and India (Wijeakumar et al., 2019)’*

“3. “Novel methods implemented in BRIGHT project”: Again, these are not novel methods. Maybe change it to “Neuroimaging methods ...”?”

We appreciate this point, which was made in both reviews and have rephrased this throughout this paper to emphasise that the implementation of these methods in a new setting is novel, not the methods themselves. The specific subheading referred to above was changed as below.

*‘Methods implemented in BRIGHT project: fNIRS, EEG and ET’*

“4. fNIRS section – *Dark skin and hair*: It’s interesting to know that the color of our skin and our hair have an impact on fNIRS signals. I wonder if the authors have ever tested this with empirical data. How different could the results be between participants with different skin colors, i.e., how big could the effect be? This could be an important question for other projects targeted on different populations.”

We appreciate the interest in this issue, which was raised in both reviews. We addressed it in response to comments in the first review on the inclusion/exclusion rates brought about by darker skin and hair. As noted there, we found it important to highlight that while more light is absorbed by dark skin and hair (this is reflected by a lower offset in the mean raw intensity readings throughout the session), all measures obtained in our tasks are relative changes in oxy-/deoxygenated blood from a reference state or baseline. Therefore, while overall more light is absorbed, task-related changes will remain largely unaffected.

“5. EEG section: The authors claimed that “the most commonly used approach lies in the examination of ERPs”. I don’t know if people will all agree with this statement. Apparently, ERP is one of the most commonly utilized EEG approaches, but I am not sure if it is fine to say it is the most. In the same section, “The vast majority of developmental EEG and ERP research ... Western ...” I am not sure this is accurate either: there are other places in the world other than Western and African countries, e.g., a large number of EEG/ERP studies have been conducted and published with children in East Asia. Maybe rephrase it as “There is scarcity of ... in the literature regarding ... in Africa or low-income countries”?”

We see the point made about the majority of papers using ERP as a mode of analysis for EEG data. We rephrased this slightly.

*‘EEG signals can be analysed in a number of ways, one commonly used approach lies in the examination of event-related potentials (ERPs).’*

We appreciate the comment on the prevalence of EEG/ERP studies in East Asian settings. To aid clarity, we changed this as below.

*‘The majority of developmental EEG and ERP research has been conducted in high-income countries. Despite previous successful implementations of this technique in the study of child development in sub- Saharan Africa ( Kihara et al., 2010), there remains a scarcity of literature using these methods to study infant neurodevelopment in low- and middle-income settings’*

# Peer review: Reviewer response for version 1

**DOI:** 10.21956/gatesopenres.14056.r27092

**Published:** 2019-05-13

**Authors:** John P. Spencer, Samuel H. Forbes

**Affiliations:** 1 School of Psychology, Lawrence Stenhouse Building, University of East Anglia (UEA), Norwich, UK; 2 School of Psychology, Lawrence Stenhouse Building, University of East Anglia (UEA), Norwich, UK

The authors present an outline of a series of methods used to collect data in the Gambia, West Africa, and that can be potentially generalised to collect detailed infant data in low- and middle-income countries. The methods used combine eye-tracking, EEG, and fNIRS, and can successfully be matched to existing data collected in the UK for cross-cultural analysis. These methods have been tested extensively, and are now being applied in Bangladesh. This article makes a potentially important contribution to the literature as a guideline for future examinations into infant cognitive development in low-resource settings, and the authors are to be commended for successful implementation of a cross-cultural assessment on this scale, and under varying conditions. However, there are a few concerns that prevent us from recommending the article for indexing at this stage.

1. Is it technically correct to define the methods presented in the paper as “Novel methods implemented in BRIGHT project?” To aid clarity, it might be prudent to consider rephrasing this in such a way as it does not look like the three methods are new ways of collecting data, rather than established ways of collecting data adapted in a novel way to new settings.
2. This might be data presented elsewhere, but Figure 1.c could be presented more clearly. It would aid display if axes were attached here, and perhaps another way could be found of demonstrating significant effects that is clearer to the reader, if this is indeed necessary. In this figure (as in the top panel of Figure 4) a higher res or larger image would be welcome for the reader.
3. As this is a methods paper, detail could be added at certain points. As an example of this, in fNIRS maintenance there is mention of local staff being trained to notice warning signs. What are these warning signs? Are there power level thresholds that you used for replacement? This would be helpful information for future studies of this type, especially as you mention how important this is for longitudinal work. Similarly on the headgear, an indication of how much more likely parts were to require replacement, and perhaps the causes here would be useful for future researchers.
4. Since this paper is set out to be a guideline for future research, an indication of the decision making process is important, especially in light of the fact that you mention certain fNIRS channels have to be disregarded due to braids etc. What are the thresholds for inclusion/exclusion? Does one have to be more tolerant in signal processing than in other settings? Each of these data collection techniques require a series of decisions about data handling, participant inclusion, signal processing etc., and the article could benefit from detail on this front.  
5. The authors mention using the eye-tracker at a lower sampling rate to improve data quality; is this based on in-lab experience, or justified through other means? Hessels *et al.* (2015 ^1^) found that the TX300 ran at 120Hz and was better than 300Hz for data loss, but I am not aware of research suggesting (even) lower is better. Figure 7 gives helpful detail of the kinds of feedback used, but again, information regarding decisions about participant dropping, retesting etc. would be welcome here.
6. The ‘acceptance of methodologies’ section is welcome information in this article, but it might be useful to add here (or perhaps earlier) information on issues with familiarisation to the lab environment, and methods, amount of time, and difficulties; here would be informative. Are there cases where an infant won’t wear an fNIRS/EEG cap at all, for example?
7. As a minor point, tasks for the fNIRS methods are mentioned but only explained further down. Perhaps an effort could be made to make this clearer for the reader?

We confirm that we have read this submission and believe that we have an appropriate level of expertise to confirm that it is of an acceptable scientific standard, however we have significant reservations, as outlined above.

## References

[1] : Qualitative tests of remote eyetracker recovery and performance during head rotation. *Behav Res Methods*.2015;47(3) : 10.3758/s13428-014-0507-6 848-59 10.3758/s13428-014-0507-6 25033759

# Response

**Published:** 2019-08-01

**Authors:** Laura Katus

**Affiliations:** University College London, UK

“1. Is it technically correct to define the methods presented in the paper as “Novel methods implemented in BRIGHT project?” To aid clarity, it might be prudent to consider rephrasing this in such a way as it does not look like the three methods are new ways of collecting data, rather than established ways of collecting data adapted in a novel way to new settings.”

We appreciate the reviewers concern regarding the emphasis here. We have gone through the manuscript and rephrased this, so that it becomes clear that the methods themselves are established and that the novelty lies in their implementation in a novel setting. We changed the subheading mentioned above to ‘ *Methods implemented in BRIGHT project: fNIRS, EEG and ET’*

“2. This might be data presented elsewhere, but Figure 1.c could be presented more clearly. It would aid display if axes were attached here, and perhaps another way could be found of demonstrating significant effects that is clearer to the reader, if this is indeed necessary. In this figure (as in the top panel of Figure 4) a higher res or larger image would be welcome for the reader.”

We thank the reviewers for their helpful suggestions to make data displayed in this figure clearer to the reader. We have taken on board their suggestions and have attached the axes labels and changed the indication of significant changes in Figure 1c. We have further amended Figures 1c and 4 to increase resolution.

“3. As this is a methods paper, detail could be added at certain points. As an example of this, in fNIRS maintenance there is mention of local staff being trained to notice warning signs. What are these warning signs? Are there power level thresholds that you used for replacement? This would be helpful information for future studies of this type, especially as you mention how important this is for longitudinal work. Similarly on the headgear, an indication of how much more likely parts were to require replacement, and perhaps the causes here would be useful for future researchers.”

We welcome the suggestions for additional detail and added them to the fNIRS section of the manuscript under ‘Maintenance of the system’.

*‘A process was implemented to ensure that key staff based in The Gambia were trained to detect NTS ‘warning signs’ when collecting data so the UK based team could be immediately contacted. These included the identification of (i) channels exhibiting unusually low raw intensity readings in reference to the values expected for the NTS system and (ii) channels showing oscillatory patterns inconsistent with the underlying physiology (i.e. heart rate or from other biological origin). The former was usually brought about by either an optode unclipping from the array, therefore affecting the readings in the channels defined by that optode and the neighbouring ones, whereas the latter was a consequence of eye tracker light being picked up by the fNIRS detectors. Staff were trained to address both of these issues by reattaching any loose optodes prior to the recording session  and checking the fit of the headgear to ensure light from the eye tracker would not corrupt the signal (for further detail on this issue see section on ‘Simultaneous fNIRS/ET recording’ below). In addition to these checks, power level checks of all sources were performed in the beginning of the project to establish a baseline and then regularly repeated. Sources evidencing significant drops between such  measurements were monitored and replaced at the next possible opportunity to prevent data loss.*

*Further, staff were trained to note signs of possible material damage to the headgear, such as stretchmarks or cracks in the silicone. Necessary spare parts were provided and stored on site, so that repairs could be carried out immediately via remote support from the system suppliers. This process has allowed local staff to detect, address and resolve all problems with the NTS fNIRS system to date, thus preventing delays and costly visits from specialists. This has been of particular importance, as in longitudinal, age-based design, adherence to timelines is essential to prevent missing data. Overall, none of the materials used had to be replaced more frequently than in previous studies, when accounting for the higher testing load.‘*

“4. Since this paper is set out to be a guideline for future research, an indication of the decision making process is important, especially in light of the fact that you mention certain fNIRS channels have to be disregarded due to braids etc. What are the thresholds for inclusion/exclusion? Does one have to be more tolerant in signal processing than in other settings? Each of these data collection techniques require a series of decisions about data handling, participant inclusion, signal processing etc., and the article could benefit from detail on this front.”

Again, we thank the reviewers for these very helpful suggestions. We added detail in the fNIRS section under ‘Dark skin and hair’.

***Dark skin and hair.*** *One issue we anticipated was the increased light attenuation in dark-skinned subjects with frequently braided hair. Dark skin did not affect the signal to noise ratio on our data during the initial pilot phase of the project ( Lloyd-Fox et al., 2014). The power of the light sources for the NTS optical imaging system we are using is 3.0 mW for the 780 nm and 4.5 mW for the 850 nm, both of which adhere to the laser safety standards for these systems ( Everdell et al., 2005). In The Gambia, from around one year of age, many of the girls start to have their hair tied or braided which creates visible artefacts in the data ( Figure 4). Where this was an issue, we asked mothers to undo the braids and ties for the duration of the fNIRS assessment. Whenever this was found to be impossible, we were still able to collect data from surrounding channels, which were not affected by hair to measure responses in some of the neighbouring regions of interest. The specific criteria varied between paradigms, according to hypotheses regarding localisation and lateralisation of the responses. Wherever studies were replications of previous studies in the UK, the same criteria for inclusion and exclusion of datasets were applied. Between the two parallel sites in the UK and The Gambia, the same criteria were applied, and no higher leniency had to be applied at the Gambian site. One important consideration in this context is that even though darker skin and hair can increase the noise of a measurement, this influence is constant across the entirety of the session and does not differ between baseline and task phase, or between conditions. As fNIRS measures relative changes in oxy-and deoxygenated haemoglobin changes, these relative measures are uniformly affected under these conditions. Similarly, as infants were assessed longitudinally, age-related changes were less affected by these issues.*

“5. The authors mention using the eye-tracker at a lower sampling rate to improve data quality; is this based on in-lab experience, or justified through other means? Hessels *et al. *(2015 ^1^) found that the TX300 ran at 120Hz and was better than 300Hz for data loss, but I am not aware of research suggesting (even) lower is better. Figure 7 gives helpful detail of the kinds of feedback used, but again, information regarding decisions about participant dropping, retesting etc. would be welcome here.”

This was based upon our own experience in the labs at Birkbeck, Kings and Cambridge. We are likewise not aware of any empirical data supporting this decision, although our understanding of the mechanism by which data loss is reduced at lower sampling rates suggested to us that it would be effective.

Under ideal conditions the differences may be small, but in this project we were not able to exercise “lab” levels of control over the ambient lighting in the testing rooms (particularly in The Gambia). Tobii do not publish the internal workings of their image processing algorithms but our working assumption is that the light sensitivity of the eye tracker’s CCD at the faster “shutter speeds” required at higher sampling rates is such that additional visual noise is acquired in the eye image. If we are correct in this assumption, then lower/less-deal ambient would compound this issue. We were also very aware that many of the testing team in The Gambia were not eye tracking experts, and given the general challenges with calibrating and tracking infants we wanted to make it as straightforward as possible to collect analyse-able data. The downsides are, of course, the attendant drop in temporal resolution (from 8ms to 16ms when moving from 120Hz to 60Hz). Given that the Gap/Overlap is the only one of our tasks for which RT is the main dependent variable, and considering the large condition and age-related differences we observe in RTs for this task, we took the decision that lower temporal resolution was, on balance, a worthwhile compromise given the expected reduction in dropout from analysis that is at the end of the data quality chain.

*‘Our experience in eye tracking with infants is that there is a trade-off between sampling rate and data quality (lost samples, accuracy and precision). For this reason we set the sampling rate of the eye trackers to 60Hz. This reduces the temporal precision of each sample of data to 16ms (vs 8ms at 120hz or 4ms at 300Hz). Our understanding of the mechanism behind this trade-off is that faster sampling rates mean less light hitting the eye tracker’s sensor on each sample, leading to a poorer quality eye image. Given that we could not exercise complete control over ambient lighting levels, particularly in the Gambia, we decided that it was worth suffering the loss of temporal resolution in order to a) make the process of calibrating and tracking the infants as easy as possible for non-eye tracking experts; and b) to maximise data quality, thus minimising dropout from analysis and longitudinal retention.’*

“6 . As a minor point, tasks for the fNIRS methods are mentioned but only explained further down. Perhaps an effort could be made to make this clearer for the reader?”

We thank the reviewers for this comment. To make it clearer to the reader that the specific adaptations made for each tasks will be discussed we included a note indicating that the detail on this will be provided in a subsequent section.

*‘The fNIRS studies implemented in the BRIGHT project assess a range of cognitive functions, including social cognition ( Lloyd-Fox et al., 2017), habituation and novelty detection (HaND, Lloyd-Fox et al., 2019), working memory ( Begus et al., 2016) as well as functional connectivity between cortical areas (for an example of the adaptations to stimuli used in these tasks please see sub-section on Stimuli below).’*
